# Metabolic reprogramming promotes Staphylococcus aureus serum resistance

**DOI:** 10.1099/mic.0.001710

**Published:** 2026-06-02

**Authors:** Samuel J. Fenn, Edward J.A Douglas, Ruth C. Massey

**Affiliations:** 1 School of Microbiology, University College Cork, Cork, Ireland; 2 APC Microbiome, University College Cork, Cork, Ireland; 3 School of Cellular and Molecular Medicine, University of Bristol, Bristol, UK; 4 Centre for Bacterial Resistance Biology, Imperial College London, London, UK; 5 Department of Infectious Disease, Imperial College London, London, UK

**Keywords:** dihydrolipoamide dehydrogenase, metabolism, serum resistance, *Staphylococcus aureus*

## Abstract

*Staphylococcus aureus* is a leading cause of bloodstream infections causing an estimated 300,000 deaths worldwide. Using a functional genomics approach, our group previously identified that adaptation of *S. aureus* to human serum is polygenic, with a clinically occurring non-synonymous mutation (V76I) in dihydrolipoamide dehydrogenase (*lpdA1*/*pdhD*) improving bacterial survival. In this work, we establish that improved serum survival of strains expressing PdhD V76I is underpinned by enhanced resistance to host-derived antimicrobials, including antimicrobial peptides and host-defence fatty acids which are prevalent in the bloodstream. Here, we demonstrate that the PdhD V76I variant has enhanced diaphorase activity, with both clinical and laboratory strains expressing this variant recycling NADH to NAD^+^ without using respiration, leading to a decrease in membrane potential. Expression of PdhD V76I conferred increased resistance to gentamicin, hydrogen peroxide, LL37, HNP-1 and arachidonic acid. However, strains which utilize PdhD V76I do not display growth defects typical of persisters and small colony variants (SCVs), with decreased NADH accumulation in serum leading to enhanced glycolysis/TCA cycle activity, improving bacterial replication in human serum. Whilst establishment of SCV and persister populations is a key survival strategy in the bloodstream, this work demonstrates how intermediate phenotypes can also be effective at promoting survival in this hostile environment. This highlights the heterogenous nature of *S. aureus* adaptation to the host environment, with an improved fundamental understanding of these processes required to allow for the development of novel therapeutics which target this process of host adaptation.

## Impact Statement

The establishment of a bloodstream infection requires the bacteria to be able to withstand the antibacterial factors found there. As one of the major global causes of these types of infections, *S. aureus* utilizes diverse strategies to survive and replicate in the bloodstream. This can include modifications to either the cell envelope or core metabolic processes such that the host’s immune features are withstood. While extreme adaptations to metabolism can lead to the major growth defects associated with persisters and small-colony variants (SCVs), here, we describe a clinically relevant adaption, where metabolism was altered such that the bacteria could survive and replicate in serum without altering the growth characteristics of the bacteria. Mutation of the gene encoding the dihydrolipoamide dehydrogenase (DLD) enzyme (*lpdA1*/*pdhD*) enhanced its diaphorase (DP) activity such that it could recycle NADH to NAD^+^ without using respiration. This led to a reduction in membrane potential that conferred resistance to gentamicin, hydrogen peroxide, LL37, HNP-1 and arachidonic acid (AA) and human serum but did not affect growth. This work uncovers a novel means of adaptation to the bloodstream by *S. aureus* and highlights how its plasticity underlies its success as a major human pathogen.

## Data Availability

The authors confirm that the data supporting the findings of this study are available with the article and supplementary material. Bacterial strains developed as part of this study are available upon request.

## Introduction

*Staphylococcus aureus* is an important human pathogen responsible for over 1 million deaths a year [1]. This opportunistic pathogen associates with humans as a commensal, with transition to a pathogenic state responsible for a wide range of diseases ranging from soft tissue to urinary tract infections [2]. The most dangerous of these diseases is bacteraemia, with *S. aureus* bloodstream infections killing in 20–30% of cases causing an estimated 300,000 deaths per year [3,5]. *S. aureus* survival and persistence in the bloodstream is a key component to progression of this disease. This opportunistic pathogen gains access to the bloodstream through initial colonization of other sites or through intravenous medical devices. Once in the bloodstream, this pathogen can disseminate to all body parts with the resulting sepsis often leading to multiple organ failure [2,6]. The ability of this pathogen to form biofilms and establish intracellular reservoirs, SCVs and persister cells increases antimicrobial tolerance and complicates treatment of *S. aureus* once an infection has embedded [7,9].

Initial establishment of an infection at a host site is mediated by an extensive arsenal of virulence factors and defensive mechanisms. Toxins, proteases and lipases secreted by *S. aureus* promote local cellular damage and liberate nutrients from the host environment [10]. Immune evasion strategies such as production of Spa/Sbi block antibody binding, with proteins such as Eap, SCIN and Ecb inhibiting complement activation [6]. This enables *S. aureus* to evade the action of the host immune system and persist in the host environment. Infection of the bloodstream by *S. aureus* is heavily dependent on defensive mechanisms, with invasive diseases such as bacteraemia or lung infections generally accepted to result in downregulation of virulence factor production [11,12]. These defences are required as the bloodstream is a heavily protected niche with multiple components exhibiting anti-staphylococcal action, such as antimicrobial peptides (AMPs) and host-defence fatty acids (HDFAs) [13,16]. The ability of *S. aureus* to overcome these host defences is critical to the establishment of a bloodstream infection, with this organism capable of adapting and evolving in-host to resist these anti-staphylococcal factors.

One key mechanism of adaptation in hostile environments is modulation of metabolism [17]. Invasion of the bloodstream naturally leads to decreased growth capacity of *S. aureus*, with the innate immune system locking away key metabolites such as iron and copper by chelating them from the environment in a process known as nutritional immunity [18,19]. This leads to respiratory dysfunction and results in naturally higher levels of tolerance by host-derived antimicrobials. *S. aureus* is also capable of producing SCVs, with mutations which negatively impact the respiratory chain (*pbgS*/*hemB*, *menD*) commonly identified *in vivo* [7,20, 21]. These SCVs exhibit slow growth due to decreased metabolic activity, increased antimicrobial tolerance and lower toxicity, leading to reduced antibacterial activity of both the immune system and antibiotics [7]. Similarly, alterations in central carbon metabolism also decrease susceptibility to host and exogenous antimicrobials, with interruption of the TCA cycle promoting survival in these hostile host environments [22,23].

This work builds upon previous work conducted in our group detailing the application of a genome-wide association study (GWAS) to identify polymorphisms associated with altered survival in human serum [24]. One of the novel effectors identified in this study was the DLD *lpdA1*/*pdhD*. PdhD re-oxidizes dihydrolipoamide to lipoamide on the E2 subunit of the pyruvate dehydrogenase complex. This enables the pyruvate dehydrogenase complex to continue producing acetyl CoA from pyruvate, feeding into both the TCA cycle and fatty acid synthesis [25]. A Valine76Isoleucine amino acid substitution in PdhD was identified to drastically improve survival of three *S. aureus* bacteraemia isolates in serum, whilst *pdhD* deletion resulted in decreased resistance to serum, suggesting that a gain-of-function mutation had occurred [24]. In this work, we demonstrate that the V76I mutation metabolically reprogrammes *S. aureus* through improved redox cycling of NADH to NAD^+^. This confers some SCV-associated phenotypes without compromising the growth of *S. aureus*, with strains expressing the PdhD V76I mutant demonstrating increased resistance to host and exogenous antimicrobials. As a result, these strains can persist and grow in human serum, demonstrating that the establishment of SCVs or persister cells is not the only pathway to survival of *S. aureus* in the bloodstream.

## Methods

### Bacterial strains, plasmids and culture conditions

Bacterial strains and plasmids used in this study are listed in Table 1. *S*. *aureus* strains were routinely maintained on Tryptic soy agar (TSA) and cultured in Tryptic soy broth (TSB) with pooled human serum added at 25% (v/v) where indicated. *Escherichia coli* strains were routinely maintained on Lysogeny broth agar and cultured in LB (Miller). For protein overexpression, *E. coli* strains were cultured in Terrific Broth (TB). When required, antibiotics were added at concentrations of 10 µg ml^−1^ for erythromycin and chloramphenicol, 90 µg ml^−1^ for kanamycin and 100 µg ml^−1^ for carbenicillin.

**Table 1.** Strains, plasmids and oligonucleotides used in this study

Strain	Description	Reference
***S. aureus* strains**		
JE2	USA300; CA-MRSA, type IV SCCmec; lacking plasmids p01 and p03; wild-type strain of the NTM	[53]
SH1000	MSSA; 8325-4 strain repaired in *rsbU* (*rsbU*+)	[54]
Newman	MSSA; strain isolated from case of secondary tubercular osteomyelitis	[55]
MRSA252	MRSA; CC30 reference strain.	[56]
JE2 *pdhD*::tn	*pdhD* transposon mutant in JE2.	[53]
Newman *pdhD*::tn	*pdhD* transposon mutant in Newman.	This study
SH1000 *pdhD*::tn	*pdhD* transposon mutant in SH1000.	This study
ASARM4	CC30 clinical bloodstream isolate, *pdhD* wild-type.	[57]
ASARM170	CC30 clinical bloodstream isolate, *pdhD* wild-type.	[57]
EOE45	CC30 clinical bloodstream isolate, *pdhD* wild-type.	[57]
ASARM161	CC30 clinical bloodstream isolate, *pdhD* V76I.	[57]
ASARM190	CC30 clinical bloodstream isolate, *pdhD* V76I.	[57]
EOE225	CC30 clinical bloodstream isolate, *pdhD* V76I.	[57]
JE2 *ndhC*::tn	*ndhC* transposon mutant in JE2.	[53]
***E. coli* strains**		
NEB5-alpha	*F−*, *φ80dlacZΔM15*, *Δ(lacZYA-argF)U169*, *deoR*, *recA1*, *endA1*, *hsdR17(rk−*, *mk+)*, *phoA*, *supE44*, *λ−*, *thi1*, *gyrA96*, *relA1*	NEB
IM08B	SA08BΩPN25-hsdS (CC8-1) (SAUSA300_0406) of NRS384 integrated between the *essQ* and *cspB* genes	[27]
NiCo21 (DE3)	*F– ompT gal dcm lon hsdSB(rB–mB–) λ(DE3 [lacI lacUV5-T7p07 ind1 sam7 nin5]) [malB+]K-12(λS) glmS6ala slyD-CBD arnA-CBD*	[58]
**Plasmids**		
**Plasmid name**	**Description**	**Reference**
pCN34	Multi-copy *E. coli S. aureus* shuttle vector. Possesses Gram-positive replicon PT181. Used for complementation. Kan^R^.	[33]
p*pdhD*	Complementation plasmid with *pdhD* under control of the *pdhA* promoter. Kan^R^.	This study
pSNP	Complementation plasmid with *pdhD* SNP (V76I AA) under control of *pdhA* promoter. Kan^R^.	This study
p*ndhC*	Complementation plasmid with *ndhC* under control of the native promoter. Kan^R^.	This study
pSK67	pTOPO type vector for protein overexpression. Under control of a T7 promoter and IPTG inducible. AmpR	[59]
pSK*pdhD*	N-terminally hexahistidine tagged PdhD overexpression plasmid.	This study
PSK*pdhD*V76I	N-terminally hexahistidine tagged PdhD V76I overexpression plasmid.	This study
**Oligonucleotides**		
SF092	ATAGTCGACGAAGAAGACGCAATTAAGAAGTCTG	
SF093	CAATTGGGAAATCTCCAACTACCATTCAATTCACCATACCTTTCCC	
SF094	GGGAAAGGTATGGTGAATTGAATGGTAGTTGGAGATTTCCCAATTG	
SF095	ATAGGTACCGCCGTAGCATATGCTACAGC	
SF055	ATAGAGCTCATGCATCACCATCACCATCATGTAGTTGGAGATTTCCCAATTG	
SF056	ATAGAATTCGCCGTAGCATATGCTACAGC	
SF125	ATAGTCGACGGGATACAAATTGATATCAATAACG	
SF126	ATAGGTACCCTTCAAATTGTACAACAATAAAGCCC	

For growth time-course experiments, *S. aureus* strains were cultured in TSB or RPMI (Gibco) amended with 1% casamino acids (RPMI-C). Growth curves were performed in 96-well plates with 200 µl of medium per well, with *S. aureus* inoculated at an initial OD_600nm_ of 0.01. Plates were incubated at 37 °C with shaking at 250 r.p.m., with OD_600nm_ monitored using a Tecan Pro200 plate reader. Growth curves were performed in the absence of antibiotics.

### DNA manipulations, mutant construction and complementation

Plasmid minipreps and PCR purifications were performed with QIAprep spin mini kit (Qiagen). PCR products for cloning applications amplified with Phusion polymerase (Thermo). Restriction digest, ligations and transformation were performed according to standard protocols. Oligonucleotide sequences (Sigma) used in this study are in Table 1.

The *pdhD*::tn mutation from JE2 was transduced into SH1000 using φ11 as performed by Krausz and Bose [26]. As *pdhD* is the last gene in the *pdhABCD* operon, we placed *pdhD* under control of the native *pdhA* promoter in our complementation plasmids. The P*_pdhA_* promoter, *pdhD* coding sequence and downstream terminator regions were amplified from MRSA252 with primers SF092/93 and SF094/95, respectively. P*_pdhA_* and *pdhD* DNA sequences were joined by overlap extension PCR through a 21 bp overlapping region on SF093 and SF094. The P*_pdhA_-pdhD* fragment was then ligated into the *E. coli*/*S. aureus* shuttle vector pCN34 through digestion with KpnI and SalI. Ligations were transformed into *E. coli* IM08B, purified and then electroporated into the required *S. aureus* strains [27]. For generation of the *pdhD* V76I complementation plasmid, the same process was followed with the *pdhD* coding sequence and terminator region amplified from ASARM161. Throughout the manuscript, pCN34 is denoted as pEmpty to demonstrate plasmid-only control.

### Serum survival assays

Pooled human serum was purchased (Clinisciences), aliquoted and stored at −20 °C until use. *S. aureus* strains were cultured in TSB O/N, normalized to 1×10^7^ c.f.u. ml^−1^ in PBS, and 20 µl was inoculated into 180 µl of 25% human serum diluted in PBS. The bacteria were then incubated at 37 °C with shaking, with three samples sacrificed per timepoint for bacterial enumeration. The same number of bacterial cells was inoculated into PBS, diluted and plated as a control. Serial dilutions were plated on TSA to determine c.f.u. ml^−1^. Survival was determined as the percentage c.f.u. in serum relative to the initial inoculum (0 h timepoint). Experiments were repeated three times with three technical replicates per timepoint.

### Antimicrobial killing assays

Cationic AMP killing assays with LL37/HNP-1 (Clinisciences) and hydrogen peroxide were performed in 1 ml of PBS with a 1×10^6^* S. aureus* inoculum. Both LL37 and HNP-1 were added at 5 µg ml^−1^ and incubated at 37 °C with shaking for 4 h. Hydrogen peroxide was added at 20 mM and incubated at 37 °C with shaking for 90 min. One millilitre of Dey–Engley broth was added to hydrogen peroxide killing assays to neutralize and prevent further killing during sample processing. Killing assays with AA (Sigma), gentamicin (Sigma) and daptomycin (Med-Chem Express) were performed in 1 ml TSB with a starting inoculum of 1×10^7^ cells. AA and gentamicin were added at 200 µM and 5 µg ml^−1^, respectively, followed by incubation at 37 °C for 2 h. Data presented as the base Log^10^ of the raw c.f.u. data. The same number of bacterial cells was inoculated into PBS, diluted and plated to act as a control.

### Laurdan membrane fluidity assay

Laurdan dye (MedChem) was used to assess membrane fluidity as previously described by Wenzel and colleagues [28]. *S. aureus* strains were cultured overnight in TSB and sub-cultured into 5 ml TSB supplemented with 1.25 mM CaCl_2_, 0.5 mM MgCl_2_ and 0.2% glucose (w/v) the following day. The bacteria were incubated for 3 h at 37 °C, normalized to an OD_600nm_ of 0.4 in the same media (pre-warmed to 37 °C) and stained with 10 µM of Laurdan dye for 5 min. Cells were washed three times in pre-warmed PBS supplemented with 1.25 mM CaCl_2_, 0.5 mM MgCl_2_ and 0.2% glucose, with samples incubated in a dry water bath set to 37 °C during processing. The cell suspension (200 µl) was added to opaque black 96-well plates (Costar), and membrane fluidity was determined by measuring fluorescence (excitation 330 nm; emission 460 and 500 nm) using a Tecan PRO200 Infinite plate reader. Generalized polarization value was calculated using the following formula: GP=(I_460_−I_500_)/(I_460_+I_500_), where I_460_ and I_500_ are the emission intensities at 460 and 500 nm, respectively. DMSO (1%) and benzyl alcohol (1%) were added to JE2 as negative and positive controls, respectively.

### PdhD overexpression and purification

The *pdhD* gene was amplified from MRSA252 (wild-type) and ASARM161 (V76I) using primer pair SF055/56 modified with a hexahistidyl tag and EcoRI/SacI restriction sites. The modified *pdhD* DNA fragments were cloned into the IPTG-inducible plasmid pSK67 using the EcoRI/SacI restriction sites, forming plasmids pSK*pdhD* and pSK*pdhD*V76I. Plasmid insert sequences were confirmed by Sanger sequencing (Eurofins). Vectors were transformed into *E. coli* NiCo21 (DE3) and selected for with carbenicillin. Single colonies were selected and cultured in LB broth for 18 h at 37 °C. Strains were then sub-cultured to an OD_600nm_ of 0.05 in TB and grown to an OD of 0.6–0.8. Cultures were then cooled to 20 °C for 45 min and induced with 0.2 mM isopropyl-*β*-d-thiogalactopyranoside. Following 18 h of culture at 20 °C, cell pellets were harvested by centrifugation and stored at −70 °C for at least 1 day prior to processing. For purification of soluble PdhD and PdhD V76I, immobilized metal ion affinity chromatography (IMAC) followed by buffer exchange with a PD-10 desalting column was used. Frozen pellets were defrosted and resuspended in lysis buffer consisting of 50 mM Tris-HCl (pH 8.0), 150 mM NaCl, 10 mM imidazole, 1.2 µg ml^−1^ lysozyme and a protease inhibitor cocktail (Thermo Scientific). Pellets were resuspended at 1 g of cell paste per 10 ml of lysis buffer. Cells were lysed with a sonic dismembranator with a 15 s/15 s ON/OFF cycle for 10 min, and samples were stored in ice throughout the sonication process.

IMAC was performed using a HiTrap Chelating HP column (Cytiva) charged with NiCl_2_. The column was equilibrated with 10 column volumes (CVs) of buffer A containing 50 mM Tris-HCl (pH 8.0), 500 mM NaCl, 10 mM imidazole and 5% (v/v) glycerol with a P1 peristaltic pump (Cytiva). Samples containing PdhD and PdhD V76I were applied to the equilibrated column at 1 ml min^−1^. Soluble PdhD and PdhD V76I were obtained with a stepwise imidazole gradient with 5 CV washes at concentrations of 10, 20, 40 and 80 mM imidazole. Samples were then eluted with 3 CV of buffer A containing 200 and 500 mM imidazole collected at 2.5 ml aliquots. PdhD samples were then analysed by SDS-PAGE for homogeneity. Pure samples were pooled and concentrated using a NeoSpin centrifugal concentrator (10 kDa cutoff). Concentrated PdhD were then applied to PD-10 desalting columns (Cytiva) and eluted with 20 mM Tris-HCl (pH 8.0), 150 mM NaCl, and reconcentrated to 10 mg ml^−1^. Samples were aliquoted, flash-frozen in liquid nitrogen and stored at −70 °C for use as required. Prior to freezing, 5% glycerol was added as a cryoprotectant. After defrosting, samples were resuspended to 2.5 ml in 50 mM Tris-HCl (pH 8.0), 150 mM NaCl, and glycerol was removed by buffer exchange with a PD-10 column.

### DLD enzyme assay

DLD activity was assayed as described previously [29] with minor modifications. The reactions were performed in 96-well plates with a final reaction volume of 250 µl. The initial reaction mixture contained 100 mM potassium phosphate buffer (pH 7.8), 1 mM EDTA, 0.4 mM dihydrolipoic acid (DHL), 0.3 mM NAD^+^ and 0.003 mM of PdhD. Reactions started upon the addition of DHL and were monitored through NADH production at A_340nm_. Reactions containing all components except DHL were used as blanks. For kinetic parameter quantification, variable concentrations of DHL and NAD^+^ were used between 0 and 20 mM. When DHL was varied, the reaction was saturated with 20 mM NAD^+^. When NAD^+^ was varied, the reaction was saturated with 20 mM DHL. Kinetic constants (V_max_, K_m_ and K_cat_) were determined using non-linear regression in GraphPad Prism (version 10.4.1).

### DP enzyme activity

DP activity of PdhD variants was measured using thiazolyl blue tetrazolium bromide (MTT) as done previously [30,31]. The reactions were performed in clear flat-bottomed 96-well plates with a final reaction volume of 250 µl. The initial reaction mixture contained 100 mM potassium phosphate buffer (pH 7.6), 0.4 mM MTT, 0.3 mM NADH and 0.003 mM of PdhD. Reactions started upon addition of MTT and monitored through production of the blue-purple coloured formazan following reduction of MTT at A_560nm_ using a Tecan Infinite 200 plate reader. For kinetic parameter quantification, variable concentrations of MTT and NADH were used between 0 and 10 mM. When MTT was varied, the reaction was saturated with 10 mM NADH. When NADH was varied, the reaction was saturated with 10 mM MTT. Kinetic constants (V_max_, K_m_ and K_cat_) were determined using non-linear regression in GraphPad Prism (version 10.4.1).

### NADH: NAD^+^ ratio determination

NADH: NAD^+^ ratio was quantified using a colourimetric assay kit (Novus) according to the manufacturer’s instructions. Samples were prepared by mechanically lysing 1×10^8^* S. aureus* cells after 16 h of culture in 3 ml of TSB or TSB with 20% human serum. Cells were resuspended in 500 µl assay buffer, and 0.1 g of glass beads (Sigma) was added to sample tubes. Mechanical lysis was performed at 6 M s^−1^ for 20 s and repeated three times using an MP FastPrep tissue homogenizer with samples stored on ice throughout. Prior to NAD^+^/NADH quantification, samples were passed through a 3 kDa centrifugal concentrator to remove protein contaminants. Colourimetric change was measured in clear flat-bottomed 96-well plates with a Tecan Infinite 200 plate reader. Figures are represented as the base log_10_ of the calculated NADH: NAD^+^ ratios.

### Membrane polarity measurement

Membrane polarity was measured using 3,3′-dipropylthiadicarbocyanine iodide [DiSC_3_(5), Thermofisher Scientific] as described previously [32] with minor modifications. Samples were cultured in 3 ml TSB or TSB with 20% human serum diluted to an OD_600nm_ of 0.4 in prewarmed (37 °C) TSB. To black flat-bottomed 96-well plates, 200 µl of culture was added per well, and DiSC_3_(5) was added to a final concentration of 1 µM. Cultures were mixed with dye and then incubated statically at 37 °C for 5 min. Fluorescence was then measured using a Tecan Infinite 200 plate reader (excitation 622 nm; emission 670 nm). Fluorescence values were divided by OD_600nm_ measurements to normalize to each sample's cell density.

### Alpha-ketoglutarate dehydrogenase activity assay

Alpha-ketoglutarate dehydrogenase activity was assessed using a colourimetric assay kit (Novus) according to the manufacturer’s instructions. Samples were prepared by mechanically lysing 1×10^8^* S. aureus* cells after 16 h incubation in 3 ml TSB. Mechanical lysis procedure was identical to that for NADH: NAD^+^ ratio determination (above). Prior to activity assessment, lysate was filtered through a 10 kDa centrifugal concentrator to remove any excess *α*-ketoglutarate and pyruvate (which interfere with the assay). Protein-containing samples were resuspended in 20 mM Tris-HCl (pH 7.4), 150 mM NaCl.

### Haemolysis assay

Ethical approval for drawing and using human blood was obtained from the Clinical Research Ethics Committee (CREC) of the Cork Teaching Hospitals [CREC reference no. ECM 4 (c) 10/12/2024 and ECM 3 (hhh) 10/12/2024]. Erythrocytes were isolated from heparinized venous blood collections from healthy volunteers. Erythrocytes were washed twice in sterile saline (0.9% NaCl) and centrifuged at 600 ***g*** for 10 min. Erythrocytes were diluted to 2% in PBS, and 100 µl was incubated with 100 µl of bacterial supernatant in a 96-well plate for 30 min at 37 °C. Plates were centrifuged for 5 min at 400 ***g***, supernatants were transferred to a sterile 96-well plate, and erythrocyte lysis was evaluated by determining the absorbance at 404 nm. Saline and 0.5% Triton X-100 were used as negative and positive controls, respectively.

## Results

### DLD mutation increases *S. aureus* serum resistance

**Fig. 1.**
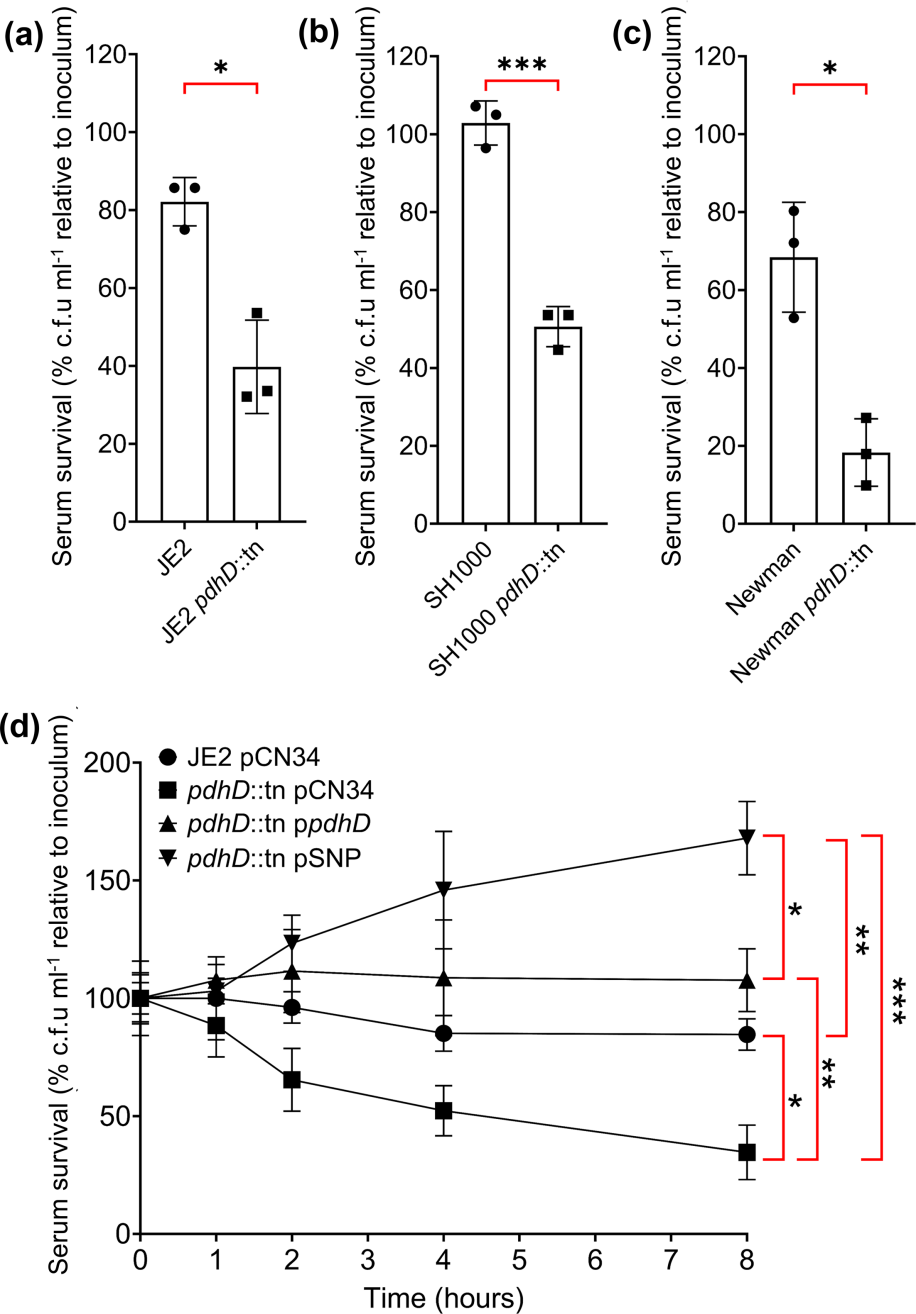
Expression of the PdhD V76I variant enhances *S. aureus fitness* in human serum. Loss of *pdhD* reduced survival of JE2 (**a**), SH1000 (**b**) and Newman (**c**) in human serum after exposure for 4 h. (**d**) Time-course of *S. aureus* JE2, *pdhD* mutant and complemented strains’ c.f.u. ml^−1^ recovery in human serum. Human serum concentration at 25% (v/v) diluted in PBS. For panels a to c, points represent independent experiments, bars represent the average, and error bars represent sd. Percentage c.f.u. recovery calculated relative to initial inoculum plated at 0 h. For panel d, points represent the average of three experiments, with error bars indicating sd. Significance determined as *<0.05, **<0.01 and ***<0.001, calculated through paired Student’s two-tailed t-test (**a–c**), and two-way ANOVA with Tukey’s (**d**) multiple comparisons test.

**Fig. 2.**
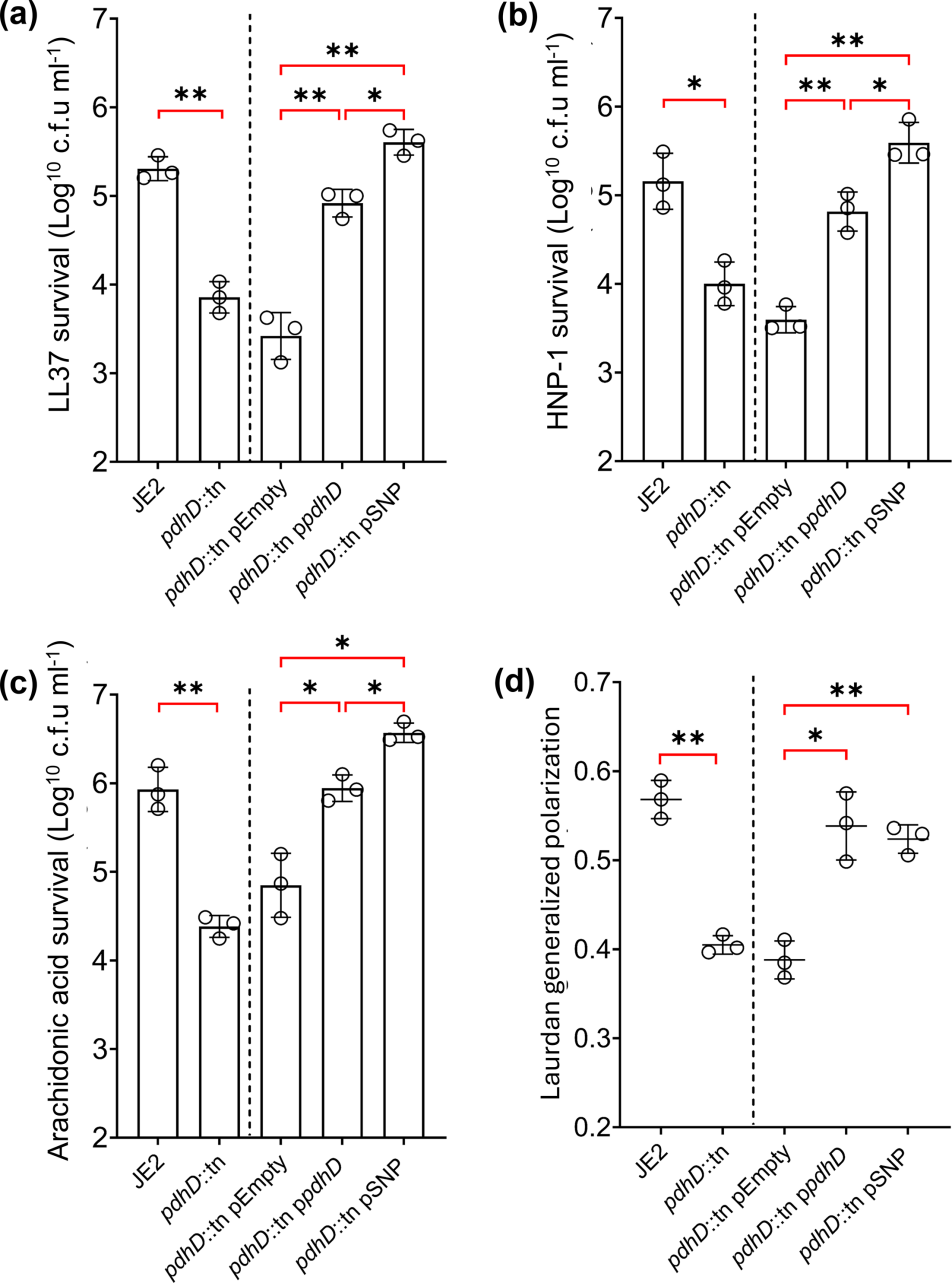
PdhD V76I increases CAMPs and HDFA resistance without impacting cytoplasmic membrane fluidity. *S. aureus* killing assays with LL37 (**a**), HNP-1 (**b**) and AA (**c**). (**d**) Membrane fluidity assessed with Laurdan dye. Dotted line: strains lacking the plasmid not statistically compared to strains with plasmids. LL37 and HNP-1 killing assays conducted in PBS at 5 µg ml^−1^ for 4 h with a starting inoculum of 1×10^6^ cells. AA killing assays conducted in TSB at 200 µM and 2.5 µg ml^−1^ for 2 h with a starting inoculum of 1×10^7^ cells. Laurdan dye added at 10 µM for 10 min followed by washing, with emissions detected at 440 and 490 nm used to infer the fluidity of the plasma membrane. Each dot represents an individual experiment with a minimum of three technical replicates. Bars and lines represent mean values, and error bars represent the sd. Significance determined as *<0.05, **<0.01, ***<0.001 and ****<0.0001 calculated by paired Student’s t-test (strains left of dotted lines) and one-way ANOVA with Dunnett’s T3 multiple comparisons test (strains right of dotted line). Non-significant findings are denoted by absence of brackets.

Human serum contains a diverse array of antimicrobial compounds which work synergistically to maintain blood sterility [13,16]. In a previously performed GWAS screening for genes which impact survival in serum, a single V76I amino acid substitution in PdhD was associated with improved survival in serum. However, the physiological basis for this was not explored (Fig. S1, available in the online Supplementary Material) [24]. To validate the previous findings, we first compared how loss of *pdhD* impacts serum survival in both MRSA and MSSA backgrounds. Strains lacking *pdhD* demonstrate decreased survival in human serum, with JE2, SH1000 and Newman *pdhD*::tn mutants exhibiting a 43, 52% and 50% reduction in c.f.u. recovery, respectively (Fig. 1a–c). This demonstrates that the *pdhD*::tn phenotype is not JE2-specific. To isolate the effect of the V76I substitution, we created a pCN34-based complementation plasmid with *pdhD* transcription under control of the native *pdhA* promoter [33]. The PdhD amino acid sequences between MRSA252 and ASARM161 are identical apart from the V76I mutation, enabling accurate assessment of PdhD V76I mutant impact. To construct both wild-type and V76I allele expressing complementation plasmids, a sequential approach was taken. The *pdhA* promoter region was amplified from the CC30 wild-type strain MRSA252 and cloned into pCN34. Next, the *pdhD* sequences from MRSA252 and ASARM161 (PdhD V76I) were amplified and inserted downstream of the P*_pdhA_* promoter, creating plasmids p*pdhD* (wild-type *pdhD*) and pSNP (mutant *pdhD*). The p*pdhD* and pSNP vectors were then electroporated into the MRSA strain JE2, and two MSSA strains, SH1000 and Newman, harbouring the *pdhD*::tn mutation and screened for their comparative fitness in serum. Restoration of the wild-type *pdhD* allele restored serum resistance to wild-type levels. Complementation with PdhD V76I increased the fitness of *S. aureus* and fostered active growth in serum after 2 h of incubation in both JE2 and SH1000 isogenic strains (Figs 1c and 2). This confirms the effect of the V76I mutation identified during the serum resistance GWAS [24].

### Strains expressing PdhD V76I are more resistant to host-derived antimicrobials

Our group previously demonstrated that loss of *pdhD* compromised survival in serum [24], whilst we have now established that the V76I substitution in PdhD increases serum resistance (Fig. 1c). To determine if this mutation provides a general protective effect or is specific to a subset of serum-derived antimicrobials, we screened susceptibility to cationic AMPs and HDFAs, with these antimicrobials acting as a barrier to establishment of bloodstream infections.

Strains lacking *pdhD* demonstrate enhanced susceptibility to the AMP LL37 and HNP-1, with a 1.4 and 1.7 log reduction in c.f.u./ml recovery as compared to wild-type, respectively (Fig. 2a, b). This is in line with the enhanced killing of *pdhD* mutants in serum (Fig. 1c). Complementation of a *pdhD* mutant with the native *pdhD* allele increases *S. aureus* resistance to LL37 and HNP-1, whilst complementation with the V76I PdhD variant provides a significant further increase in protection against both AMPs (Fig. 2a, b). A similar trend is observed when testing the killing of these strains by AA, with loss of *pdhD* sensitizing * S. aureus* to AA, whilst complementation with the V76I substitution enhanced *S. aureus* resistance when compared to native *pdhD* complementation (Fig. 2c). Clinical strains from the original serum survival screen expressing PdhD V76I were then screened for susceptibility to AMPs and HDFAs. *S. aureus* isolates harbouring this mutation demonstrate increased resistance to LL37, HNP-1 and AA when compared to a random selection of CC30 isolates (Fig. S3). This confirms the biological significance of the naturally occurring V76I mutation and demonstrates that this allele provides a generic protective effect against serum-derived antimicrobials. Increased resistance to these antimicrobials enables strains expressing the PdhD V76I to quickly initiate replication in serum, explaining the identification of this allele in our previously performed GWAS [24].

Acetyl-CoA produced by the pyruvate dehydrogenase complex feeds into both the TCA cycle and straight-chain fatty acid synthesis, with the latter dictating phospholipid membrane composition and fluidity. Loss of *pdhA* and the subsequent reduction in straight-chain fatty acid synthesis have been reported to increase membrane fluidity of *S. aureus* [25]. This renders strains more susceptible to attack by membrane-active antimicrobials such as LL37, HNP-1 and AA. Alterations in membrane fluidity could impact susceptibility to these antimicrobials, altering survival in serum. To determine if the membranes of strains expressing PdhD V76I are altered, we employed Laurdan labelling as a direct measure of membrane fluidity [28]. Complementation with wild-type *pdhD* and *pdhD* V76I decreased membrane fluidity to the same extent (Fig. 2d), confirming that alterations in membrane fluidity are not responsible for the increased resistance to LL37, HNP-1 and AA of the PdhD V76I-expressing strains.

### The *pdhD* SNP allele increases *S. aureus* hydrogen peroxide and gentamicin resistance without inducing an SCV phenotype

**Fig. 3.**
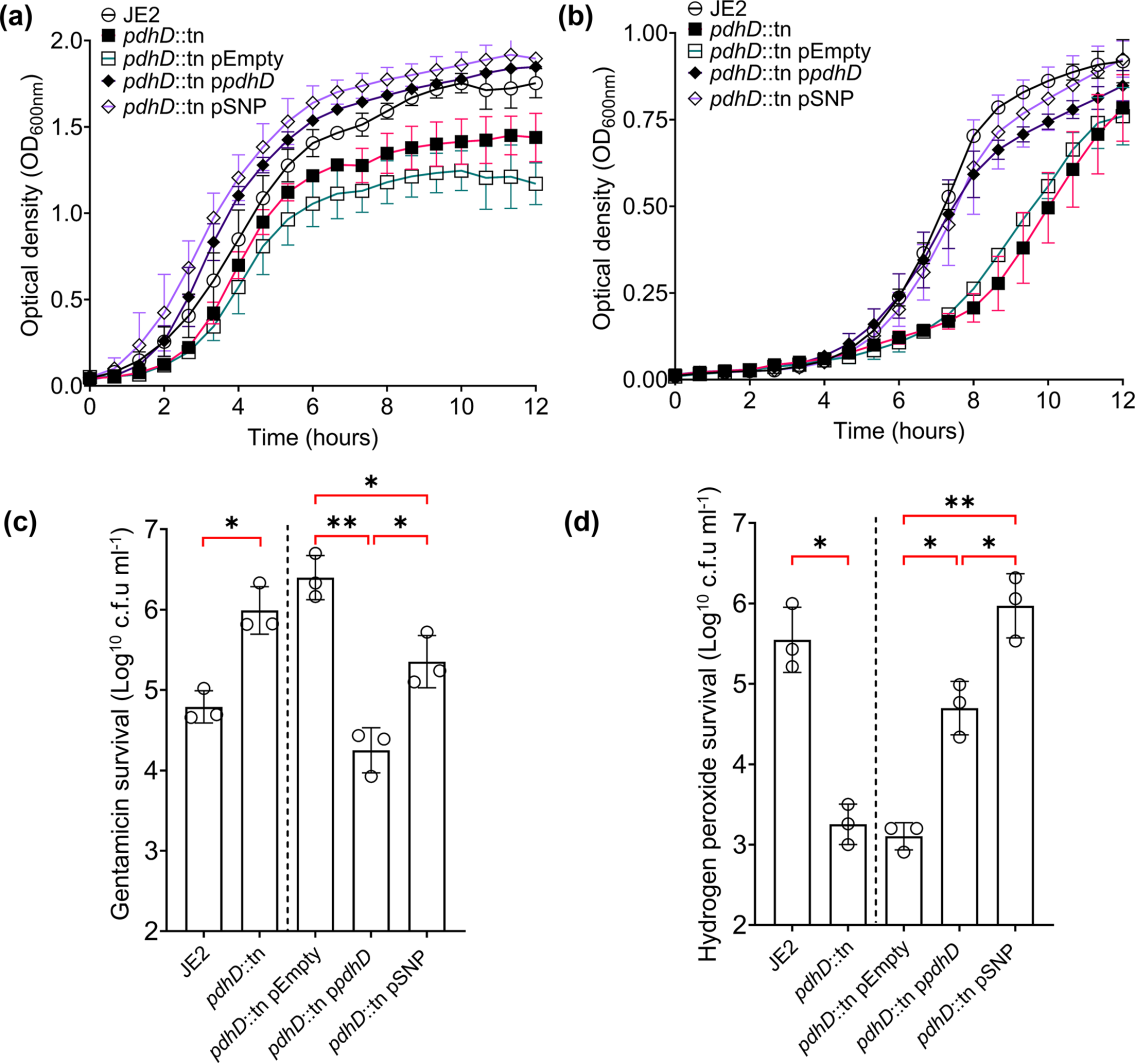
PdhD V76I mutation provides protection against gentamicin and hydrogen peroxide without compromising growth. (**a, b**) Growth curves of *pdhD* mutant and complements in TSB and RPMI-C, respectively. *S. aureus* killing assays with gentamicin (**c**) and hydrogen peroxide (**d**). Dotted line indicates strains lacking the plasmid not statistically compared to strains with plasmids. For growth kinetics, points represent the average of three independent experiments with a minimum of four technical replicates per experiment. Gentamicin and hydrogen peroxide killing assays conducted with an initial inoculum of 1×10^7^ cells. Gentamicin killing conducted in TSB at 5 µg ml^−1^ for 2 h, and hydrogen peroxide killing conducted in PBS at 20 mM for 90 min. Points represent individual experiments with a minimum of three technical replicates; bars represent the mean values, with error bars representing sd. Significance determined as *<0.05, **<0.01, ***<0.001 and ****<0.0001 calculated by paired Student’s t-test (strains left of dotted lines) and one-way ANOVA with Dunnett’s T3 multiple comparisons test (strains right of dotted line). Non-significant finding denoted by absence of bracket.

Acetyl-CoA produced by the pyruvate dehydrogenase complex is the main source of fuel for the TCA cycle, reacting with oxaloacetate to form citrate. Mutants of the pyruvate dehydrogenase complex adopt an SCV phenotype with loss of *pdhA* and *pdhB* shown to compromise replication of *S. aureus* [25]. Visual examination of individual colonies of the isogenic wild-type and *pdhD* mutant pairs and the clinical strains containing the V76I mutation verified that they were all normal in size. However, to ensure that this mutation does not alter growth, we performed growth curves in both TSB and RPMI-1% casamino acids (RPMI-C). Strains deficient for *pdhD* demonstrate decreased growth in both TSB and RPMI-C (Fig. 3a, b). Complementation with the native *pdhD* allele and *pdhD* V76I restored growth to wild-type levels in both TSB and RPMI-C, suggesting that there is no growth defect associated with the V76I mutation (Fig. 3a, b). SH1000 isogenic strains demonstrate the same pattern, whilst no growth defect is observed in clinical strains encoding PdhD V76I (Fig. S4).

Increased hydrogen peroxide and gentamicin resistance are hallmarks of SCV in *S. aureus*, with the notable exception of haem autotrophs being more susceptible to hydrogen peroxide through loss of catalase activity [34,36]. These phenotypes can also be used to screen for metabolic modifications, with killing by hydrogen peroxide and gentamicin dependant on a functional electron transport chain (ETC) [35,37]. Mutation of *pdhD* resulted in increased resistance to gentamicin, whilst loss of *pdhD* sensitizes *S. aureus* to hydrogen peroxide-mediated killing (Fig. 3c, d). Complementation with native *pdhD* enhanced gentamicin activity and improved hydrogen peroxide resistance. However, complementation with the *pdhD* SNP allele only partially increased gentamicin killing, whilst it simultaneously improved the ability of *S. aureus* to resist hydrogen peroxide when compared to the native *pdhD* allele (Fig. 3c, d). These phenotypes were confirmed with MIC assays, where *pdhD* V76I complementation increases resistance to gentamicin and hydrogen peroxide without impacting the MIC of other tested antimicrobials in both JE2 and SH1000 backgrounds (Table S1). This elevated resistance is not mediated by a growth defect, as PdhD V76I-containing strains demonstrate wild-type growth under tested conditions. Given the role of *pdhD* in central carbon metabolism and associated increases in hydrogen peroxide/gentamicin resistance, it is likely that the increased serum survival exhibited by this SNP is metabolically mediated, although not through reduced growth output.

### PdhD V76I demonstrates increased DP activity

**Fig. 4.**
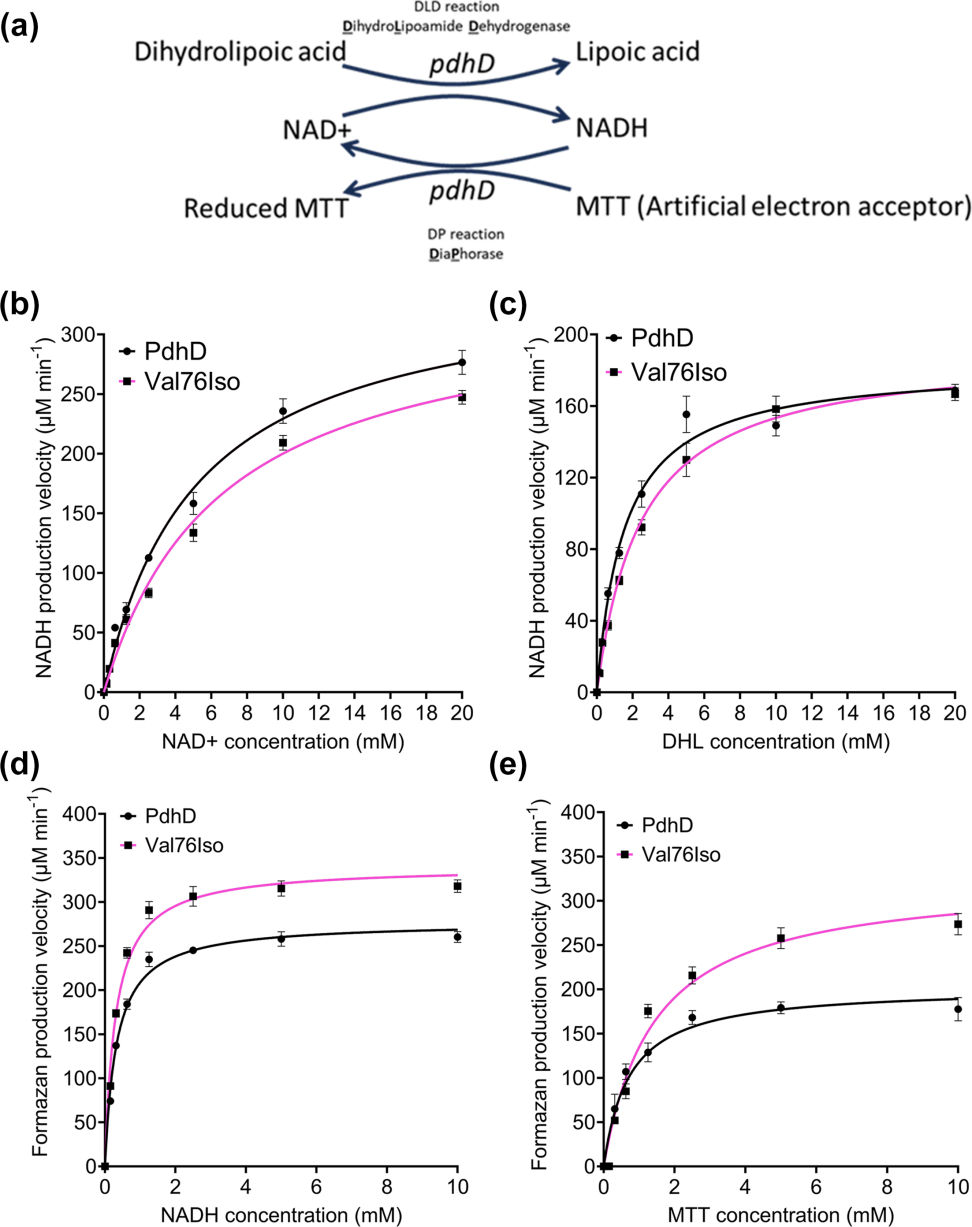
PdhD V76I mutation causes a minor reduction in DLD activity whilst increasing DP activity. (**a**) Schematic diagram outlining reaction schemes for DLD and DP assays. DLD assays performed with variable NAD^+^ (**b**) and DHL (**c**). DP assays performed with variable NADH (**d**) and MTT (**e**). All reactions conducted in 0.1 M potassium phosphate buffer pH 7.2 at 30 °C. DLD assay measured through appearance of NADH at A_340nm_. DP assay measured through production of formazan product at A_570nm_. Points represent mean of three independent experiments with error bars indicating sd. At least two technical replicates were conducted per experiment. Reaction velocities for Michaelis–Menten kinetics calculated using GraphPad Prism.

Given the increased resistance to gentamicin and hydrogen peroxide, we examined whether this mutation is associated with defective pyruvate dehydrogenase function. An interruption in central carbon metabolism would decrease TCA and ETC activity, elevating tolerance to multiple antimicrobials. PdhD catalyses two reactions, a DLD reaction, which regenerates the essential lipoate cofactor in the pyruvate dehydrogenase complex, and a DP reaction, which oxidizes NADH to NAD^+^ using an external electron acceptor (Fig. 4a) [38]. To understand the biochemical effect of the V76I on PdhD catalytic function, we purified both variants for enzymatic activity assessment.

The enzymatic kinetic parameters of the DLD reaction were characterized using a reaction consisting of NAD^+^, DHL and PdhD with production of NADH measured spectrophotometrically (Fig. 4a). Initial reaction velocities were determined at varying substrate concentrations for both NAD^+^ (Fig. 4b) and DHL (Fig. 4c). These values were used to determine kinetic parameters by non-linear regression and Michaelis–Menten kinetics. This revealed that the V76I mutation results in a small decrease in substrate affinity (K_m_) and catalytic efficiency (K_cat_/K_m_) for NAD^+^ and DHL (Table 2), indicating that this mutation decreases the ability of PdhD to bind DHL/NAD^+^ and regenerate lipoate. We next sought to characterize DP activity of this variant using a reaction consisting of NADH alongside the artificial electron acceptor MTT which is reduced to form a purple formazan product, enabling kinetic tracking of DP activity (Fig. 4a). Initial reaction velocities were determined at varying concentrations of NADH (Fig. 4d) and MTT (Fig. 4e), with kinetic parameters determined as performed for the DLD assay. PdhD V76I demonstrated enhanced DP activity when using NADH (Fig. 4d and Table S2) and MTT (Fig. 4e and Table 2) as substrates. The effect of this mutation on DP activity is more drastic than that observed for the DLD assay (Fig. 4b, c), with the V76I form of PdhD achieving higher maximum reaction rates (V_max_), elevated number of substrate molecules a single enzyme can convert into product per second (k_cat_) and increased catalytic efficiency (k_cat_/K_m_) when using NADH and MTT as substrates in comparison to the MRSA252 wild-type PdhD (Table 2).

**Table 2.** Kinetic parameters assessing activity of PdhD vs PdhD V76I. DLD assays assessed using variable concentration of NAD^+^ and DHL, whilst DP assay assessed using variable concentrations of NADH and MTT. Maximal reaction rate (V_max_), substrate affinity (K_m_), substrate molecule turnover per second (K_cat_) and catalytic efficiency (K_cat_/K_m_) calculated and compared to wild-type PdhD kinetic parameters. Kinetic parameters calculated using data presented in Fig. 4 with GraphPad Prism. Significance determined as *<0.05, **<0.01 and ***<0.01 calculated by paired two-tailed Student’s t-test. Non-significant comparisons denoted by lack of asterisks

Substrate	Parameter	Variant	
		PdhD	Val76Iso
**NAD^+^**	*V_max_ (µM min^−1^)*	348.2±21.3	331.1±23.5
	*K_m_ (µM)*	3.184±0.82	4.045±1.1
	*K_cat_ (s^−1^)*	116.1±3.78	110.4±5.14
	*K_cat_/K_m_ (µM s^−1^)*	36.46±9.55	27.29±6.64*
**DHL**	*V_max_ (µM min^−1^)*	182.8±8.95	191.6±7.35
	*K_m_ (µM)*	1.548±0.327	2.487±0.597
	*K_cat_ (s^−1^)*	60.94±2.99	63.86±2.45
	*K_cat_/K_m_ (µM s^−1^)*	39.36±6.73	25.68±6.1*
**NADH**	*V_max_ (µM min^−1^)*	277.4±8.1	340.5±11.5***
	*K_m_ (µM)*	0.3291±0.042	0.3032±0.046
	*K_cat_ (s^−1^)*	92.48±2.705	113.5±3.85**
	*K_cat_/K_m_ (µM s^−1^)*	280.93±27.15	374.34±43.7*
**MTT**	*V_max_ (µM min^−1^)*	202.6±18.3	325.6±26.1**
	*K_m_ (µM)*	0.7169±0.23	0.8218±0.41
	*K_cat_ (s^−1^)*	67.54±6.11	108.5±8.7**
	*K_cat_/K_m_ (µM s^−1^)*	94.2±22.1	129.58±12.26*

### Increased DP activity decreases *S. aureus* intracellular NADH accumulation

Based on the increased DP activity exhibited by PdhD V76I, we hypothesized that this enzyme is contributing to NADH reoxidation to NAD^+^. Maintenance of the NADH: NAD^+^ ratio is essential to metabolic function of *S. aureus* with NAD^+^ serving as an essential cofactor in the TCA cycle and NADH used by type-II NADH dehydrogenases to reduce menaquinone (MK) for use in the ETC [39]. High NADH: NAD^+^ ratios are indicative of reductive stress and lead to inhibition of the TCA cycle and respiration, whilst the inverse is true for low NADH: NAD^+^ ratios. The ability of *S. aureus* to alter its metabolism in response to stress is pivotal to its ability to colonize harsh environments such as serum.

**Fig. 5.**
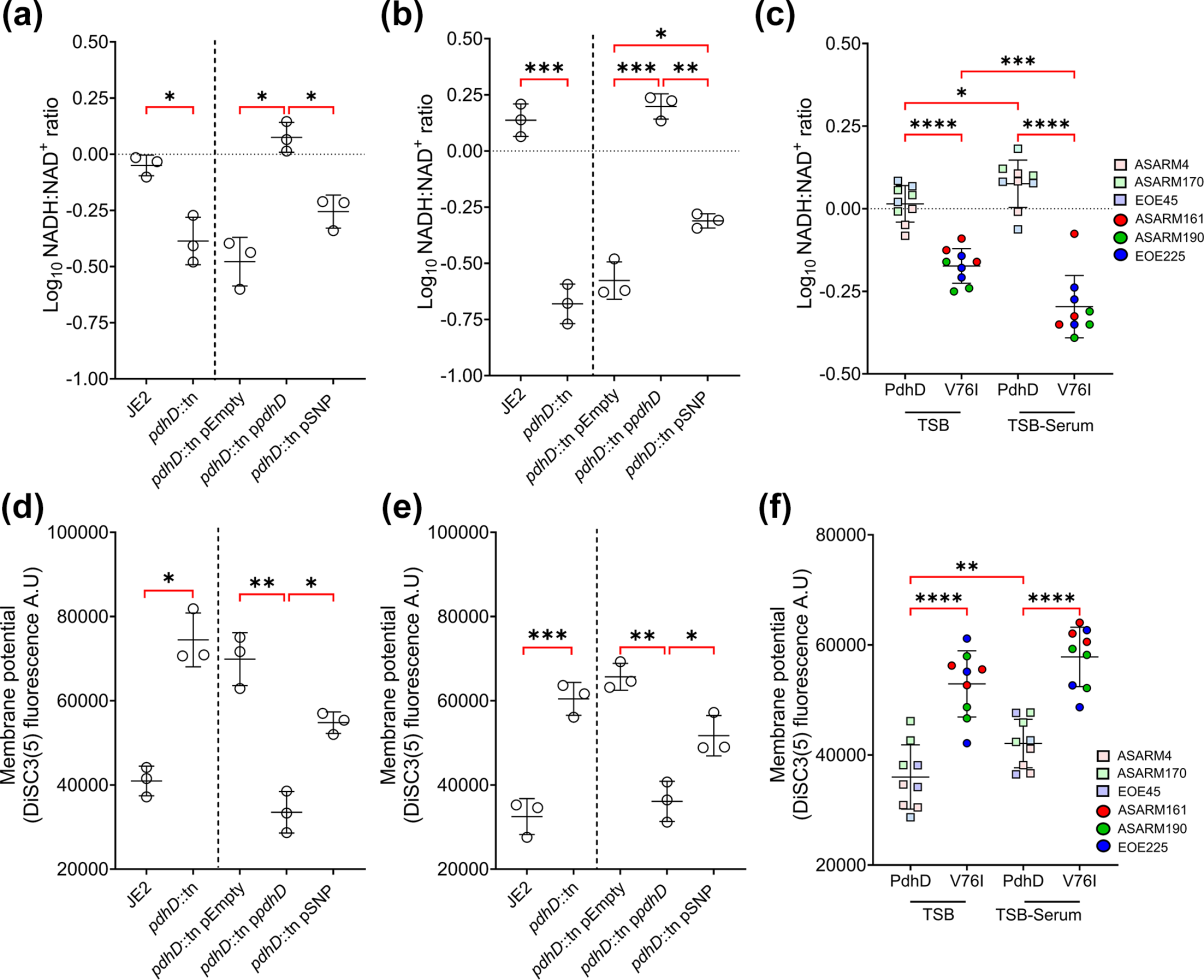
Expression of PdhD V76I is associated with decreased NADH accumulation and lower membrane potential. NADH: NAD^+^ ratio determination of isogenic *pdhD* mutants and complements in TSB (**a**) and TSB-Serum (**b**). NADH: NAD^+^ ratio determination of clinical CC30 strains containing wild-type and mutant alleles of *pdhD* (**c**). Measurement of *S. aureus* membrane potential in TSB (**d**) and TSB-Serum (**e**). Membrane potential of CC30 clinical strains expressing PdhD V76I in TSB and TSB-Serum (**f**). Dotted line indicates strains lacking the plasmid not statistically compared to strains with plasmids. Serum added at 25% (v/v) when indicated. Points represent individual experiments averaged from two technical replicates. Lines indicate the mean of these experiments with error bars denoting sd. For panels a, b, d and e, statistical significance was determined as *<0.05, **<0.01, ***<0.001 and ****<0.0001 through paired Student’s t-test (strains left of dotted line) and one-way ANOVA with Dunnett’s T3 multiple comparisons test (strains right of dotted line). Panels c and f calculated by two-way ANOVA with Tukey’s multiple comparisons test (**c, f**). Non-significant finding denoted by absence of bracket.

To determine if PdhD V76I DP activity is biologically relevant, we screened the impact of this variant on the NADH: NAD^+^ ratio of *S. aureus* when cultured in TSB and TSB-Serum. In both media, *pdhD* mutation leads to a decrease in the NADH: NAD^+^ ratio indicative of a defective TCA cycle (Fig. 5a, b). The main source of cellular NADH is the TCA cycle, producing six molecules per glucose subunit. Loss of *pdhD* inhibits the pyruvate dehydrogenase complex, preventing the formation of acetyl-CoA, starving the TCA cycle of its main carbon source [20,39]. As a result of this metabolic dysfunction, the total pool of NADH and NAD^+^ is decreased in *pdhD* mutants, with a 42.5% reduction when compared to JE2 in TSB (Fig. S5). Complementation with the native *pdhD* improved the NADH: NAD^+^ ratio in both TSB and TSB-Serum, whilst restoration of the *pdhD* SNP allele only partially complemented this phenotype (Fig. 5a, b). This indicates that strains expressing PdhD V76I have lower levels of NADH in comparison to wild-type and native complement strain. In both complemented strains, the total NADH and NAD^+^ pool is similar to wild-type, demonstrating that generation of these coenzymes is not impacted by the PdhD V76I mutation (Fig. S5). To determine if this same pattern is exhibited in the clinical strains which naturally express PdhD V76I (ASARM161, ASARM190 and EOE225), we compared the NADH: NAD^+^ ratio to representative CC30 strains (ASARM4, ASARM170 and EOE45) which encode wild-type PdhD. Consistent with the complementation data, *S. aureus* strains expressing the *pdhD* mutant allele demonstrate a lower NADH: NAD^+^ ratio when compared to wild-type (Fig. 5c). This phenotype is enhanced by the presence of serum with PdhD V76I-expressing strains further reducing the NADH: NAD^+^ ratio when compared to TSB only (Fig. 5c).

Lower NADH levels can be an indicator of decreased metabolic activity [20,40]. NADH functions as an electron donor to the ETC, serving as a substrate for type II NADH dehydrogenases. NADH dehydrogenases oxidize NADH to NAD^+^, donating two electrons to MK to form menaquinol (MKH_2_), which is used as an electron donor for cytochrome oxidases to drive proton motive force generation [39]. Therefore, lower NADH levels result in decreased membrane potential, reducing the ability of *S. aureus* to produce energy and redox cycle between NADH and NAD^+^. To confirm that membrane potential is altered in PdhD V76I-expressing strains, we employed the voltage-sensitive dye DiSC_3_(5) in both TSB and TSB-Serum. Bacteria with high negative membrane potential take up DiSC_3_(5) resulting in dye quenching, whilst strains with depolarized membranes release the dye causing an increase in fluorescence [41]. Loss of *pdhD* resulted in increased fluorescence in both TSB and TSB-Serum, indicative of lower electrogenic potential and decreased metabolism (Fig. 5d, e). Complementation with native *pdhD* increased membrane polarization, whilst strains expressing PdhD V76I also exhibit partially reduced membrane potentials, albeit not to the same extent as a *pdhD* mutant (Fig. 5d, e). We next tested the clinical strains and observed the same trend, with strains which express PdhD V76I exhibiting decreased membrane potential in comparison to representative CC30 strains (Fig. 5f). This explains increased resistance to gentamicin and hydrogen peroxide of isogenic and clinical strains expressing PdhD V76I, with membrane depolarization associated with resistance to both antimicrobials [35,37].

Reductions in membrane potential are also associated with decreased phenol soluble modulin and haemolysin secretion through repression of the Agr quorum-sensing system, decreasing haemolysis of red blood cells [42,43]. To test if expression of PdhD V76I alters haemolytic activity*,* we screened the ability of the *S. aureus* to lyse human red blood cells. Loss of *pdhD* decreased haemolysis levels to that of an *agrA* mutant indicating shut off *S. aureus* toxin production. Complementation with native *pdhD* increases haemolysis, whilst the V76I mutant only partially compensates for loss of *pdhD*, displaying decreased haemolysis in comparison to native *pdhD* complementation (Fig. S6). This highlights how the serum resistance adaptation of strains with modified DLD function comes at the expense of virulence potential.

### PdhD V76I DP activity decreases proton flux through NADH dehydrogenases, reprogramming *S. aureus* metabolism

**Fig. 6.**
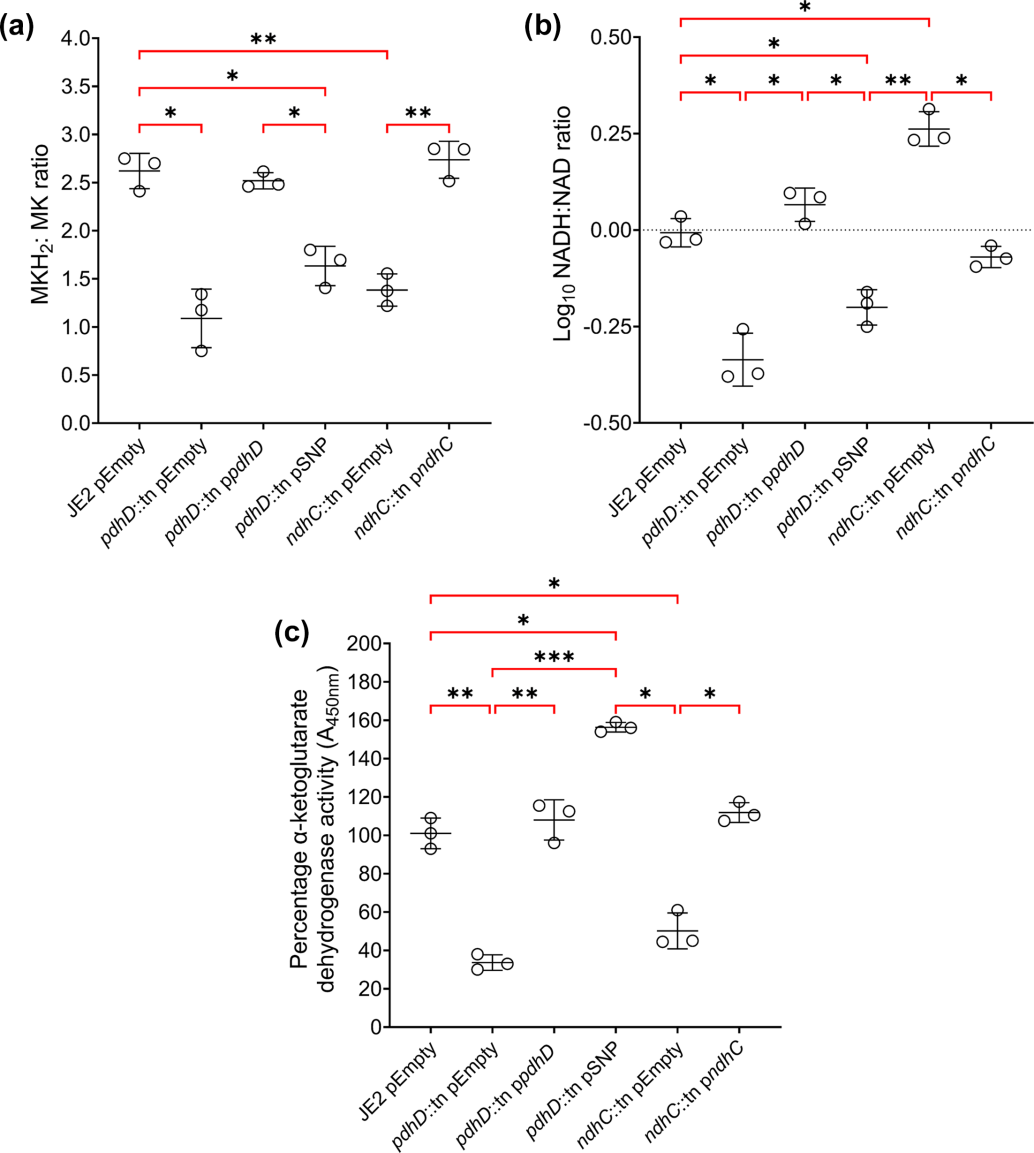
Metabolic reprogramming by PdhD V76I variant simultaneously reduces the levels of NADH and MKH_2_, impairing oxidative phosphorylation and promoting substrate-level phosphorylation. (**a**) Ratio of MKH_2_: MK and (**b**) NADH: NAD^+^ in *pdhD* and *ndhC* mutants plus complements. (**c**) *α*-Ketoglutarate dehydrogenase activity as a measure of TCA cycle function. Points represent individual experiments averaged from two technical replicates. Lines indicate the mean of these experiments with error bars denoting sd. Significance determined as *<0.05, **<0.01 and ***<0.001, calculated by one-way ANOVA with Dunnett’s T3 multiple comparisons test. Non-significant finding denoted by absence of bracket.

Whilst PdhD V76I demonstrates a reduction in DLD activity *in vitro* (Table 2), this effect is small and does not explain the decreased membrane potential. NADH is used by the type II NADH dehydrogenases to reduce membrane-bound MK. If PdhD is reducing NADH more efficiently, less will cycle through NADH dehydrogenases decreasing *S. aureus* membrane potential and reducing the menaquinol: MK ratio (MKH_2_: MK). To determine if this is true, we compared the MKH_2_: MK and NADH: NAD^+^ ratios of our *pdhD* mutant and complemented strains to that of the NADH dehydrogenase mutant *ndhC*::tn. As previously reported, loss of *ndhC* leads to a lower MKH_2_: MK ratios and a higher NADH: NAD^+^ ratio as the protons from NADH cannot be used to reduce MK in the bacterial membrane, leading to NADH accumulation (Fig. 6a, b) [39]. Complementation with *ndhC* under control of its native promoter restored the ratios of both redox pairs (Fig. 6a, b). Loss of *pdhD* also decreased the MKH_2_: MK and NADH: NAD^+^ ratios with lower TCA cycle and ETC activity inhibiting production of NADH, lowering MK reduction rates (Fig. 6a, b). Restoration of the wild-type *pdhD* allele restored the MKH_2_: MK ratio and NADH:NAD^+^ ratio to that of JE2 pEmpty. Complementation with the *pdhD* SNP allele did not restore the MKH_2_: MK ratio, whilst the NADH: NAD^+^ ratio was drastically decreased as previously seen (Fig. 6a, b). In terms of the MKH_2_: MK ratio, strains lacking *ndhC* and expressing PdhD V76I demonstrate similar defects in MK reduction (Fig. 6a). This decrease in the MKH_2_: MK for strains expressing PdhD V76I suggests that less NADH is being re-oxidized by NdhC for MK reduction, with PdhD V76I contributing to NADH oxidation in the cytoplasm.

Decreased NADH flux through NADH dehydrogenases explains the intermediate resistance of PdhD V76I expressing *S. aureus* strains to gentamicin and hydrogen peroxide, with loss of *ndhC* also demonstrating resistance to these antimicrobials through decreased PMF (Fig. S7). However, NADH dehydrogenase mutants exhibit mild growth defects in media, whilst strains expressing PdhD V76I can match wild-type growth rates in liquid media. The major metabolic difference between a *ndhC*::tn mutant and strains encoding *pdhD* V76I allele is the NADH: NAD^+^ balance. One of the major products of the TCA cycle is NADH, with the accumulation of NADH inhibiting TCA enzymes such as isocitrate and *α*-ketoglutarate dehydrogenase [44]. With the level of NADH low in strains expressing PdhD V76I, TCA cycle activity would not be inhibited, enabling substrate-level phosphorylation to continue when oxidative phosphorylation (respiration) is decreased. To validate this, we assessed TCA cycle activity through quantification of *α*-ketoglutarate dehydrogenase activity in cytoplasmic preparations derived from our isogenic *pdhD/ndhC* mutants and complements. As expected, mutation of *pdhD* and *ndhC* resulted in decreased *α*-ketoglutarate dehydrogenase activity, with loss of *pdhD* downregulating the TCA cycle and deletion of *ndhC* leading to accumulation of NADH (Fig. 6c). Complementation with *pdhD* and *ndhC* wild-type alleles restored activity, whilst restoration of *pdhD* SNP allele increased *α*-ketoglutarate dehydrogenase activity by 58% in comparison to wild-type and strains which are proficient for *ndhC* (Fig. 6c). This confirms that whilst oxidative phosphorylation is moderately impaired in strains expressing PdhD V76I, enhanced substrate-level phosphorylation (TCA cycle and glycolysis) can continue through increased redox cycling of NADH to NAD^+^.

**Fig. 7.**
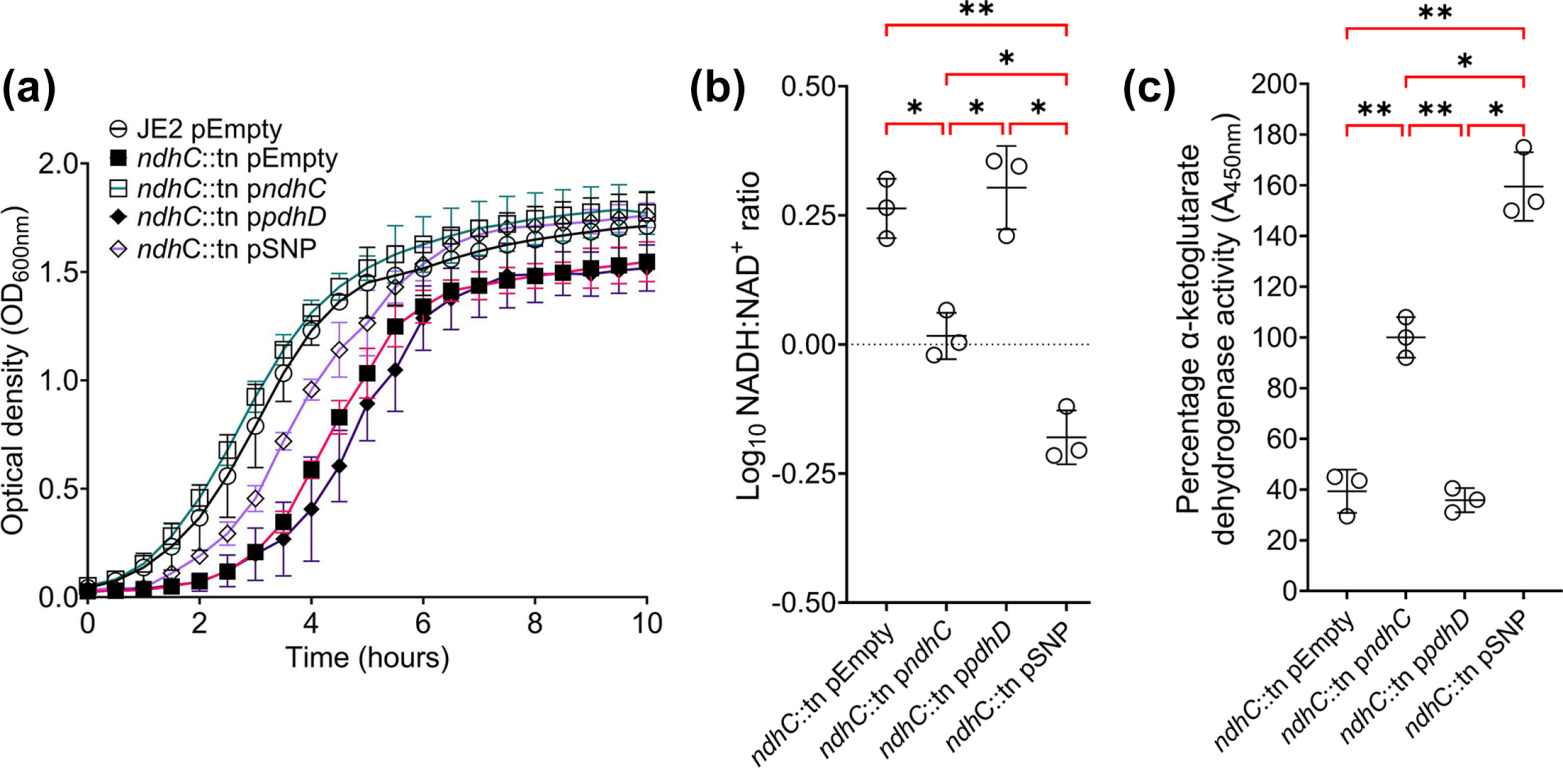
The *pdhD* SNP allele restores growth of an *ndhC* mutant through enhancing TCA cycle activity. (**a**) Expression of PdhD V76I in *ndhC* mutant background partially restores growth defect. (**b**) PdhD V76I expression in *ndhC* mutant decreases the NADH: NAD^+^ ratio (**c**) and increases *α*-ketoglutarate dehydrogenase activity. All experiments conducted in TSB only, with percentage *α*-ketoglutarate dehydrogenase activity relative to JE2 pEmpty (Fig. S8). For panel a, points represent the average of three experiments, and error bars denote sd. Optical density kinetic reads taken using a Tecan Pro200 plate reader. For panels b and c, points represent individual experiments averaged from two technical replicates. Lines indicate the mean of these experiments with error bars denoting sd. Significance determined as *<0.05, **<0.01 and ***<0.001, calculated by one-way ANOVA with Dunnett’s T3 multiple comparisons test. Non-significant finding denoted by absence of bracket.

Growth defect exhibited by *ndhC* mutants is due to shutdown of the ETC leading to accumulation of NADH, which inhibits the TCA cycle [39]. To determine if the *pdhD* V76I variant can restore growth of *ndhC* mutants, the wild-type and mutant *pdhD* complementation plasmids were transformed into *ndhC*::tn, and growth kinetics were screened in TSB. Wild-type *pdhD* overexpression does not result in restoration of growth, whilst expression of PdhD V76I variant partially decreases the growth defect associated with *ndhC*::tn (Fig. 7a). After 16 h of culture, pellets were extracted, and the NADH: NAD^+^ ratio and *α*-ketoglutarate dehydrogenase activity were assessed. Previously, loss of *ndhC* led to increased NADH accumulation (Fig. 6b). Expression of the PdhD V76I variant lowered NADH accumulation in the *ndhC* mutant background leading to increased *α*-ketoglutarate dehydrogenase activity (Fig. 7b, c). This enables partial restoration of growth in *ndhC* mutants as *S. aureus* can generate more energy through glycolysis and the TCA cycle (Fig. 7a). As a result, complementation of *pdhD* and *ndhC* mutants with the PdhD V76I variant facilitates wild-type growth in lab media as increased TCA cycle activity and glycolysis can counteract the defect in ETC activity. However, since normal ETC function is interrupted through decreased NADH dehydrogenase activity, increased resistance to host-derived antimicrobials such as hydrogen peroxide, AMPs and HDFAs enables strains expressing PdhD V76I to initiate active growth in serum faster than normal (Fig. 1d).

## Discussion

Improved survival in the bloodstream enhances the ability of *S. aureus* to sustain an infection and facilitate spread to new host sites [6]. Metabolic adaptation underpins this process, with reduction in metabolic activity employed to enable *S. aureus* to survive and adapt to this hostile environment [8,17, 20]. This decreased metabolic output promotes survival of *S. aureus* when free in blood and intracellularly within phagocytes such as neutrophils and macrophages, with the innate immune system playing a major role in controlling *S. aureus* bacteraemia [6]. The work discussed in this paper focuses on extracellular survival of * S. aureus* in human serum.

**Fig. 8.**
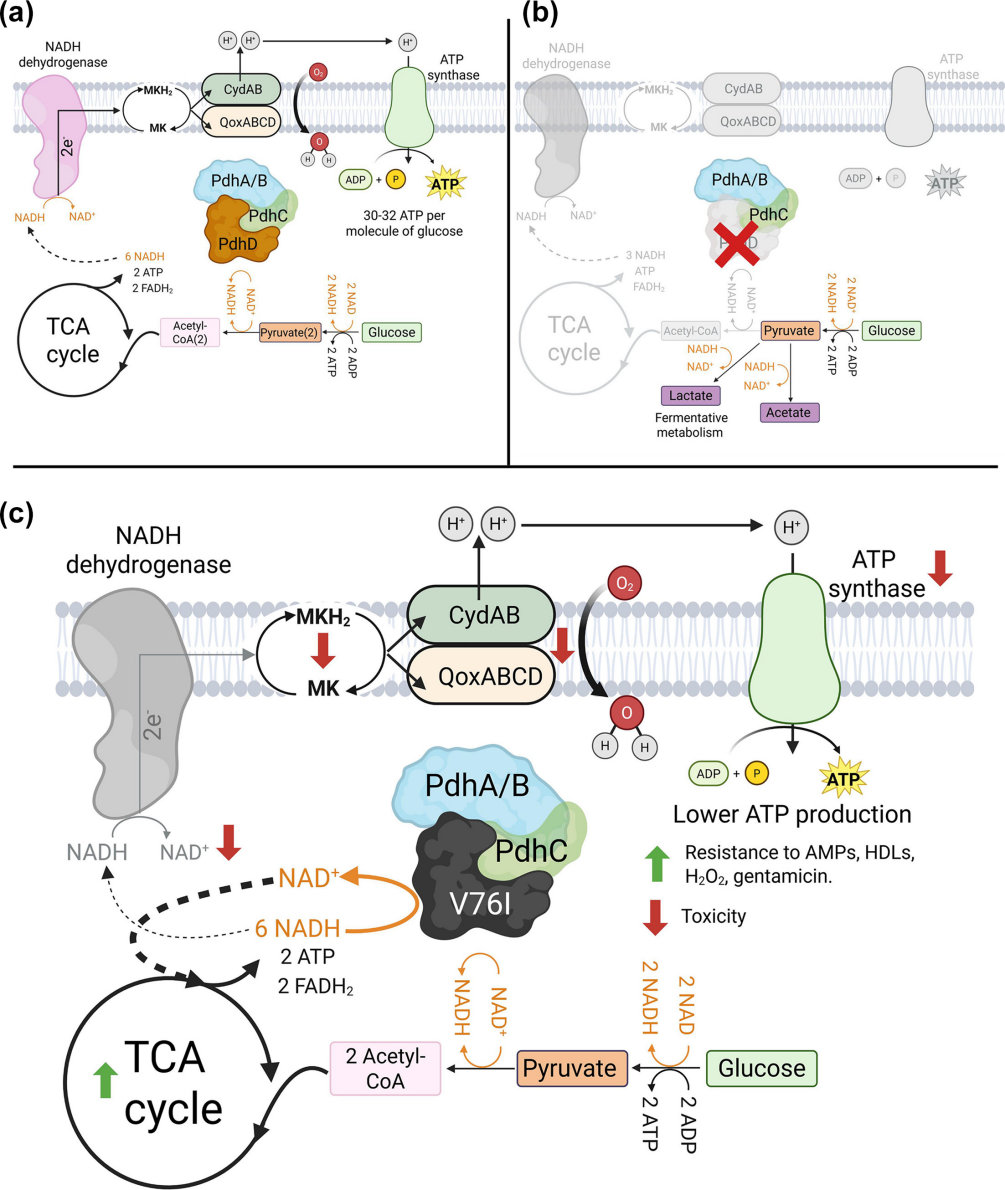
Schematic detailing metabolic outcomes for wild-type (**a**), *pdhD*::tn (**b**) and *pdhD* V76I (**c**). (**a**) Strains with a functional pyruvate dehydrogenase complex generate energy and redox cycle NADH to NAD^+^ through the ETC and oxidative phosphorylation. Glucose is converted into two acetyl-CoA molecules which enter the TCA cycle, resulting in production of six NADH molecules. NADH dehydrogenase oxidizes this coenzyme back to NAD^+^ whilst reducing MK, with this electron carrier being used by cytochrome oxidases for generation of a proton gradient and subsequent ATP production. (**b**) Strains lacking a functional pyruvate dehydrogenase complex are unable to synthesize acetyl-CoA, starving the TCA cycle of its major carbon source. As a result, less NADH is produced inhibiting NADH dehydrogenase-dependent respiration and reducing membrane potential. Pyruvate is fermented to lactic or acetic acid for generation of small amounts of ATP and to encourage limited redox cycling of NADH to NAD^+^. (**c**) Strains expressing the mutant PdhD V76I variant display enhanced DP activity, promoting redox cycling of NADH to NAD^+^. Reduction of the intracellular NADH pool limits flux through NADH dehydrogenases, reducing efficiency of the ETC. As a result, strains expressing the PdhD V76I mutant demonstrate enhanced resistance to antimicrobials. Loss of full ETC function is compensated for through increased TCA cycle activity and increased abundance of NAD^+^ encouraging glycolysis. Figures designed with BioRender with publication licences g8aqbql (**a**), ziuf2yg (**b**) and oou84jt (**c**).

As part of a previous study in which a population-based approach was employed to identify genes associated with human serum survival, a nonsynonymous amino acid substitution was identified in DLD (*lpdA1*/*pdhD*) which enhanced the fitness of three clinical *S. aureus* isolates in human serum [24]. We demonstrate that this mutation enables *S. aureus* to quickly initiate active growth in serum through alterations in NADH/NAD^+^ and MK/MKH_2_ cofactor metabolism, whilst loss of *pdhD* drastically decreased the survival of *S. aureus* in human serum. Fig. 8 presents a model for the metabolic scenarios in * S. aureus* strains with wild-type *pdhD* (A), deletion of *pdhD* (B) and encoding the *pdhD* SNP allele. In strains with a normally functioning pyruvate dehydrogenase complex, pyruvate is converted into acetyl-CoA. DLD regenerates lipoamide from dihydrolipoamide on the E2 subunit of the pyruvate dehydrogenase, enabling the complex to continuously convert pyruvate to acetyl-CoA for the TCA cycle and fatty acid synthesis. The TCA cycle produces 6 NADH, 2 ATP and 2 FADH_2_ per single glucose molecule, with the NADH produced subsequently utilized as a substrate by type II NADH dehydrogenases. The electrons from this oxidation reaction are used to reduce MK to menaquinol. This electron donor is used by cytochrome oxidases to drive the generation of proton motive force with ATP synthase producing ATP from this proton accumulation (Fig. 8a). This represents a redox-balanced energy generation system, with NADH dehydrogenase produced NAD^+^ circulating back into the TCA cycle for further NADH generation. In this scenario, *S. aureus* relies upon oxidative phosphorylation (respiration) for ATP generation yielding 30–32 ATP per glucose molecule. With complete loss of *pdhD*, *S. aureus* metabolism shuts down (Fig. 8b) [39,44, 45]. In pyruvate dehydrogenase-deficient mutants, acetyl-CoA cannot enter the TCA cycle. This starves the TCA cycle of its substrates, with a lack of NADH production reducing the ability of *S. aureus* to generate proton motive force and energy through oxidative phosphorylation. As a result, *S. aureus* relies upon glycolysis and acid fermentation to produce small amounts of energy and redox cycle between NADH and NAD^+^, causing the decreased growth rates associated with loss of *pdhD* (Fig. 8b) [20,25, 43].

The *pdhD* SNP is associated with increased survival in serum reprogrammes *S. aureus* metabolism. This V76I mutation increases the DP activity of this enzyme (Fig. 4d, e, Table 2), allowing PdhD to contribute to redox cycling of NADH and NAD^+^ (Figs5ac and 8c). Consequently, a partial reduction in ETC activity is observed, as less NADH is available for NADH dehydrogenases to facilitate the reduction of MK (Fig. 5d–f [39]). This increases *S. aureus* resistance to AMPs, HDLs, hydrogen peroxide and gentamicin, all of which rely upon a functional ETC to promote maximum damage [35,37, 46]. This resistance profile explains the ability of strains encoding the *pdhD* SNP to better tolerate serum, with AMPs and HDLs being major growth inhibition factors in this environment. In terms of a *pdhD* transposon mutant exhibiting increased susceptibility to hydrogen peroxide, interruption of glycolysis has previously been shown to reduce haem biosynthesis [36]. Mutations which perturb haem biosynthesis render *S. aureus* highly susceptible to hydrogen peroxide, as catalase is a haem-dependent enzyme [36,47]. With the pyruvate dehydrogenase complex being a major component of central metabolism linking glycolysis and TCA cycle activity, the *pdhD* mutant may be sensitized to hydrogen peroxide by a similar mechanism as observed with a reduction in glycolysis. This was not investigated further, as we concluded that the PdhD V76I variant was not an inactivating mutation.

Despite the PdhD V76I variant phenocopying *pdhD* mutant strains in exhibiting increased resistance to gentamicin, and decreased NADH, MKH_2_ and toxicity, the pathways taken to reach these phenotypes are different. Loss of *pdhD* results in metabolic shutdown, whilst strains encoding the *pdhD* SNP have redirected metabolism to favour substrate-level phosphorylation as opposed to oxidative phosphorylation (Fig. 8b, c). This encourages ATP generation through glycolysis and the TCA cycle. The ability of the PdhD V76I variant to reduce NADH means that fermentative metabolism of pyruvate to lactic and acetic acid is not required to recycle NADH to NAD^+^ (Fig. 8b [48]). This prevents acidification of the media enabling normal cellular processes to continue and the TCA cycle to continue functioning. The lack of NADH accumulation increases TCA cycle activity, as NADH is a competitive inhibitor of *α*-ketoglutarate and isocitrate dehydrogenase, whilst the increased availability of NAD^+^ enhances glycolysis with this coenzyme required for glycolytic ATP generation (Fig. 8 [44,49]). Consequently, strains carrying the *pdhD* SNP can maintain faster growth rates despite exhibiting decreased oxidative phosphorylation, whilst sustaining increased levels of resistance to antimicrobials normally associated with respiratory chain defects [20,35, 46]. This ability to restore growth is displayed when expressing PdhD V76I in both *pdhD* and *ndhC* mutant backgrounds and represents a novel strategy of host environment adaptation (Fig. 7a). Such mutations likely represent a within-host evolutionary dead-end due to associated reductions in toxicity which could inhibit colonization of both new host sites and other patients [11,50]. This is especially true of bloodstream infections, where the host is either killed or cured. The ability of *S. aureus* to colonize, establish and initially cause an infection is underpinned by its extensive repertoire of virulence factors. Downregulation or loss of toxicity is a common adaptation employed by *S. aureus* to promote survival *in vivo*, but entry to another site or host transmission will require these systems to be reactivated [2,11, 50]. Therefore, whilst this mutation promotes survival during an infection, the loss of toxicity suggests that these strains would struggle to establish a new infection.

Whilst the DLD function of this variant exhibits a small decrease in activity, it is still able to catalyse conversion from pyruvate to acetyl-CoA. This, in part, explains its distinct behaviour when compared to a strain devoid of *pdhD*, as enough acetyl-CoA can be generated for TCA cycle activity and fatty acid synthesis. Loss of pyruvate dehydrogenase activity results in increased membrane fluidity, as straight-chain fatty acids cannot be synthesized *de novo*. As a result, *S. aureus* membrane consists almost entirely of branched-chain fatty acids providing easier access to membrane-targeting antimicrobials and increasing sensitivity to AMPs, HDFAs and daptomycin [25]. The membrane fluidity of strains encoding the *pdhD* SNP is not obviously affected under the conditions tested in this work. However, we cannot rule out that the exact composition of fatty acids may have changed in the membrane or may be further altered under different physiological conditions.

Exactly how this PdhD alteration promotes this increased DP activity is unclear. The V76I mutation does not occur in the catalytic pocket or near the co-factor-binding sites of PdhD. This mutation does occur along the dimerization interface of PdhD, with DLD activity dependent on formation of a homodimer [30,51]. The increased DP activity could be linked to decreased ability to form dimers, with monomeric DLD previously shown to exhibit enhanced DP activity [31,51, 52]. Increased transition to monomeric DLD was previously shown to be driven by changes in pH; however, mutations which inhibit this dimerization also have a similar effect [30]. Any impact on PdhD dimerization is caveated by the fact that these effects have only been observed for mammalian forms of this enzyme and may not apply to prokaryotic PdhD [31,51, 52]. However, given the functional conservation of PdhD as part of the pyruvate dehydrogenase complex in both eukaryotes and prokaryotes, it is likely that dimerization efficiency plays a conserved role in regulating enzymatic activity of PdhD. As such, a small reduction in dimerization efficiency may be driving the increased DP activity exhibited by strains carrying the PdhD V76I, with this single amino acid change reprogramming * S. aureus* metabolism and promoting improved serum tolerance.

Entry and survival in human blood are significant barriers to the continuance of infection by bacteria. Mutations which promote survival in this hostile environment enable *S. aureus* to adapt faster, with persistence in this environment underpinning within-host spread to other environments. This work highlights how subtle changes in central carbon metabolism can lead to drastically improved outcomes in human serum. Whilst SCVs often result in persistent and recurrent infections, the drastic reductions in metabolic output lead to decreased growth [20,37]. Through modulation of PdhD function, *S. aureus* can sustain growth output whilst also maintaining enhanced resistance to host-derived antimicrobials normally associated with SCVs. Subtle alterations in metabolism enable *S. aureus* to persist and thrive in serum, facilitating sustained infection once in a host. This highlights how complete metabolic shutdown is not the only path to survival for *S. aureus* in the hostile host environment.

## Supplementary material

10.1099/mic.0.001710 Supplementary Material 1.

## Abbreviations

AA: arachidonic acid
AMPs: antimicrobial peptides
CREC: Clinical Research Ethics Committee
CVs: column volumes
DHL: dihydrolipoic acid
DLD: dihydrolipoamide dehydrogenase
DP: diaphorase
ETC: electron transport chain
GWAS: genome-wide association study
HDFAs: host-defence fatty acids
IMAC: immobilized metal ion affinity chromatography
MTT: thiazolyl blue tetrazolium bromide
SCVs: small-colony variants
TB: Terrific Broth
